# Using Explainable Machine Learning to Improve Intensive Care Unit Alarm Systems

**DOI:** 10.3390/s21217125

**Published:** 2021-10-27

**Authors:** José A. González-Nóvoa, Laura Busto, Juan J. Rodríguez-Andina, José Fariña, Marta Segura, Vanesa Gómez, Dolores Vila, César Veiga

**Affiliations:** 1 Cardiovascular Research Group, Galicia Sur Health Research Institute (IIS Galicia Sur), 36213 Vigo, Spain; laura.busto@iisgaliciasur.es (L.B.); cesar.veiga@iisgaliciasur.es (C.V.); 2 Department of Electronic Technology, University of Vigo, 36310 Vigo, Spain; jjrdguez@uvigo.es (J.J.R.-A.); jfarina@uvigo.es (J.F.); 3 Intensive Care Unit Department, Hospital Álvaro Cunqueiro (SERGAS), 36213 Vigo, Spain; marta.segura.pensado@sergas.es (M.S.); vanesa.gomez.casal@sergas.es (V.G.); dolores.vila.fernandez@sergas.es (D.V.)

**Keywords:** alarms, explainable machine learning, Intensive Care Unit, machine learning, MIMIC, patient monitoring, sensors

## Abstract

Due to the continuous monitoring process of critical patients, Intensive Care Units (ICU) generate large amounts of data, which are difficult for healthcare personnel to analyze manually, especially in overloaded situations such as those present during the COVID-19 pandemic. Therefore, the automatic analysis of these data has many practical applications in patient monitoring, including the optimization of alarm systems for alerting healthcare personnel. In this paper, explainable machine learning techniques are used for this purpose, with a methodology based on age-stratification, boosting classifiers, and Shapley Additive Explanations (SHAP) proposed. The methodology is evaluated using MIMIC-III, an ICU patient research database. The results show that the proposed model can predict mortality within the ICU with AUROC values of 0.961, 0.936, 0.898, and 0.883 for age groups 18–45, 45–65, 65–85 and 85+, respectively. By using SHAP, the features with the highest impact in predicting mortality for different age groups and the threshold from which the value of a clinical feature has a negative impact on the patient’s health can be identified. This allows ICU alarms to be improved by identifying the most important variables to be sensed and the threshold values at which the health personnel must be warned.

## 1. Introduction

**Figure 1:**
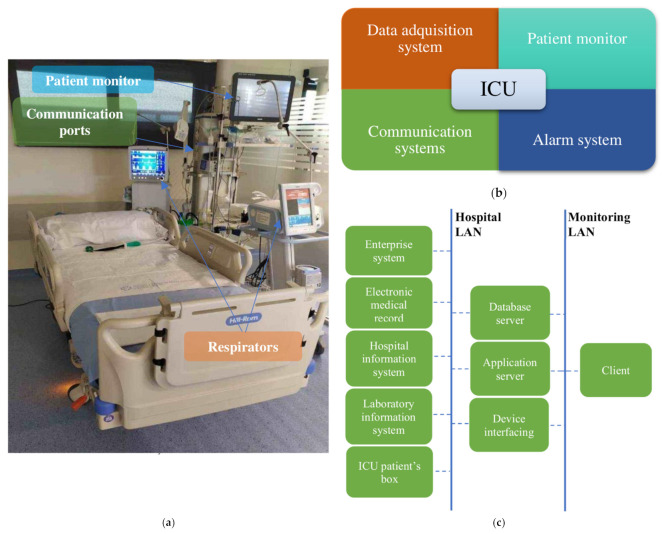
(**a**) ICU box at Álvaro Cunqueiro Hospital; (**b**) ICU monitoring blocks; (**c**) ICU communication system.

The Intensive Care Unit (ICU) is the area of the hospital where the most critical patients are located, on whom it is necessary to carry out continuous monitoring. Patient monitoring equipment in charge of acquiring the data that health personnel use for decision-making is located beside each ICU bed (also called a box). It should be noted that the concept of patient monitoring is broad. It is not limited to the information provided by the electronic devices located next to the bed, but it also covers, for example, the work of the laboratory responsible for blood test analyses, as well as the information generated by the different actuator equipment such as respirators [1]. Figure 1a shows a box from an ICU at Álvaro Cunqueiro Hospital. To monitor health variables, the architecture of the ICU monitoring system consists of four main components, shown in Figure 1b. Such systems centralize and organize patient information including admission information, vital signs, and medical notations, allowing its analysis and subsequent decision-making about patients. The first component, the data acquisition system, is responsible for real-time acquisition and storage of data from biosensors or mechanical sensors for further analysis by health personnel. The second component, the patient monitor, deals with medical monitoring screens located next to beds, which allow patients’ main physiological parameters to be visualized. The third component, the alarm system, warns health personnel whenever any monitored variable is outside a predefined safety range or some type of anomaly is detected. The last component, the communication system, is in charge of data transmission through a Local Area Network (LAN) or a Medical Information Bus (MIB/RS232). It connects all of the elements of the system for data transmission, from boxes to servers, for communication among the different areas of the hospital (such as the administrative system or the sample analysis and research laboratories) and for communication among the different hospitals that are part of the same health network. Figure 1c shows the outline of an ICU communication system.

The ICU monitoring process generates a large amount of data which is difficult to analyze manually by health personnel, so the implementation of systems that do this automatically is of great interest within the ICU [2,3,4]. Artificial intelligence has already been applied in ICU settings [2]; however, most artificial intelligence works is traditionally focused on predicting certain events within the ICU and analyzing the results obtained using different types of statistical metrics (Area Under Receiving Operating Characteristic Curve (AUROC), Precision, Recall, Specificity, and Accuracy). In recent years, however, there has been an increasing interest in the use of techniques that analyze the causes of the results obtained by the models, which are known as explainable machine learning [5]. This allows the model to stop being a “black box”, making it possible to analyze, among other things, which are the features that have the highest impact within the model. Generally, patients are considered as a unique age group, but it is well known that age affects many biologic processes, and there are many works that demonstrate the effect that patient age has on phenotypes [6] and health condition in several clinical scenarios, including neurological [7], cardiovascular [8] and many others.

Therefore, it is of major interest to extend explainable machine learning techniques to ICU massive data analysis in order to improve ICU alarm systems. The purpose of this article is to propose a methodology to automatically identify the threshold values of the clinical variables at which alarm systems must warn healthcare personnel. This methodology is based on explainable machine learning techniques which split patients into age groups instead of setting up a unique classifier for the whole dataset, which allows more precise and specific threshold values for each age group to be defined.

The remainder of the article is structured as follows. In Section 2, the materials used are detailed, namely the ICU database (MIMIC-III), predictor algorithm (XGBoost), and explainable machine learning technique (Shapley Additive Explanations, SHAP). In Section 3, the proposed pipeline is explained. In Section 4, the results are provided and analyzed. This includes the evaluation of the mortality prediction model using different statistical metrics as well as SHAP outcomes. Finally, the discussion and conclusions of the work are presented.

## 2. Materials

### 2.1. Data Source

For the realization of this work, the open access database MIMIC-III (Medical Information Mart for Intensive Care III) [9] created by MIT (Massachusetts Institute of Technology) was used. It includes information from 61,532 ICU stays at Beth Israel Deaconess Medical Center between 2001 and 2012.

The database includes information such as demographics, vital sign measurements made at the bedside (~1 data point per hour), laboratory test results, procedures, medications, caregiver notes, imaging reports, and mortality (both in and out of hospital).

### 2.2. Prediction Algorithm: XGBoost

XGBoost [10] belongs to the category of Boosting techniques in Ensemble Learning, that is, a collection of predictors that combine multiple models in order to achieve better prediction accuracy. Boosting techniques attempt to correct the errors made by previous models in successive ones via additional weighting. Unlike other boosting algorithms where the respective weights of misclassified branches are increased, in Gradient Boosted algorithms a loss function is optimized instead. XGBoost is an advanced implementation of gradient boosting with the following objective function (1), optimized at each *t* iteration.

(1) $$L^{\left(t\right)}=\overset{n}{\underset{i=1}{\sum }}l\left(y_{i},{\tilde{y}}_{i}^{\left(t-1\right)}+f_{t}\left(x_{i}\right)\right)\;+\;Ω\;\left(f_{t}\right)$$

where *l* is a differentiable convex loss function that must be transformed into another one in a Euclidean domain by using Taylor’s Theorem, the pair (*y_i_*, x_*i*_) represents the training set, ỹ_*i*_ is the final prediction, and Ω(*f_t_*) is the regularization term used to penalize more complex models through both Lasso and Ridge regularizations and to prevent overfitting.

Once this optimization is performed the algorithm builds the next learner, which achieves the maximum possible loss reduction without exploring all tree structures, but rather by building a tree greedily by applying the Exact Greedy Algorithm. This algorithm consists of three steps. It starts with a single root (which contains all the training examples); then, it iterates over all features and values per feature, evaluating each possible split loss reduction. Finally, the stop condition is checked, stopping the branch from growing if the gain for the best split is not positive; otherwise, execution continues. A more detailed explanation may be found in XGBoost’s white paper [10].

An open-source package developed by the University of Washington implements the algorithm [11]. It stands out for its ability to obtain the best results in different benchmarks, and is one of the best-optimized algorithms for computing parallelization. In addition, it has support for Graphic Processor Units (GPUs), which allows the capacity of the algorithm to be fully exploited.

Fitting XGBoost requires setting three types of parameters, namely general, booster, and learning task parameters. General parameters specify the booster used, commonly a tree or linear model. Booster parameters depend on the selected booster and define its internal configuration parameters, such as the learning ratio or the number of estimators, among others. Learning task parameters decide on the learning scenario, specifying the corresponding learning objective.

### 2.3. Shapley Additive Explanations (SHAP)

SHAP values can be used to analyze the features that have the highest impact in a prediction task, in addition to determining the threshold values from which they have a positive or negative impact in the prediction.

SHAP values use the Shapley interaction index from game theory to capture local interaction effects. They follow from generalizations of the original Shapley value properties [12] and allocate credit not just among each player of a game, but also among all pairs of players. SHAP interaction values consist of a matrix of feature attributions (interaction effects in off-diagonal terms and the remaining effects in diagonal terms). By enabling the separate consideration of interaction effects for individual model predictions, Tree Explainer can uncover notable patterns that might otherwise be missed. SHAP specifies the explanation as (2). A more detailed description is given in [12].

(2) $$f\left(x\right)=E_{X}\left(f\left(X\right)\right)+\overset{M}{\underset{j=1}{\sum }}\phi_{j}$$

where f(x) is the predictor model, x is the instance for which we want to compute the contributions, *E_X_*(*f*(*X*)) is the summatory of the mean effect estimate for each feature, and ϕ_*j*_ ∈ R is the feature attribution for a feature *j* (the Shapley value).

The code has been implemented in an open-source package developed by the University of Washington and Microsoft Research [13].

## 3. Method

In order to improve the ICU monitoring process we first sought to identify the most important variables to be included in a monitoring system for ICU patients on the basis of age by developing a three-step specific pipeline that included the aforementioned components. The first step included a pre-processing stage with two main purposes: (1) To separate patients depending on their ages and produce five different datasets, as described in Section 3.1; and (2) To pre-process the data in order to remove missing data and extract the set of features involved in the analysis. The second step was devoted to setting up a classification state by predicting patient mortality within the ICU. The last step selected the most important features based on the SHAP technology for artificial intelligence explanation.

### 3.1. Cohort Selection

The first step in the ICU monitoring pipeline included a pre-processing stage that allowed the patients suitable for the study to be identified and for each of them to be assigned to one of the four age groups. Although MIMIC-III includes data from 61,532 ICU stays, the number of actual patients is 46,476, as some of them were admitted to ICU several times. As stated in Section 1, one of the objectives sought in this work was to customize the monitoring system taking into account the patient age range. For this purpose, four patient groups were defined. Age limits, denoted as X_i_, were based on those used by the Centers for Medicare and Medicaid Services of the US Government [14], i.e., X_A_:(18, 45]; X_B_:(45, 65]; X_C_:(65, 85]; X_D_:(85, ∞]. Since the goal was to improve the monitoring system for the adult ICU, patients under the age of 18 were not part of the study.

From this initial cohort, patients with more than 50% of the temporal variables empty in the database were discharged. Table 1 shows the total number of patients remaining in each age group after data curation; this produced a dataset of 36,693 patients for all four groups.

### 3.2. Feature Extraction

Once the cohort was selected, the next step was feature extraction. In this work, 33 clinical variables were considered, specifically those that had less than 20% empty data and were frequently used in ICU analysis [15]. Data from the first 24 h of each of the selected patient’s first ICU stay were considered. Derived from those time series, the maximum, minimum, mean, and standard deviation values of the variables were obtained, except for urine output (for which only total volume was considered). This produced a total of 129 features.

The complete set of variables is shown in Table 2, where the features extracted from them are identified.

K–Nearest Neighbors was the imputation method used to fill in empty features; it is one of the most widely used imputation methods. In it, each sample’s missing value is imputed using the mean value of its k nearest neighbors. Two samples are close if the features that neither of them is missing are close. In this work, the Scikit–Learn package [16] was used.

### 3.3. XGBoost Classifier Setup for Mortality Prediction

After the pre-processing stage, the XGBoost model was trained to predict patient mortality within the ICU for each age group. This required using an outcome variable, in this case the mortality during ICU stays, because it is one of the most serious problems that should be warned of by an alarm system. It is also one of the most widely used variables in the literature [17,18].

The training and test of each XGBoost classifier were repeated for each age group *X_i_*, producing four different classifiers (*C_i_*). All age groups were split into a training subset and a test subset using the 80/20 ratio, which is frequently used in this realm; the experiment was repeated five times, with data being randomly shuffled beforehand.

As the complete dataset contained 2980 patients who died and 34,535 that survived the ICU episode, it is clearly an unbalanced situation. XGBoost was adjusted taking into account this imbalance. The XGBoost developers indicate that for these cases it is necessary to adjust the parameter in charge of the control of the balance of positive and negative weights, useful for unbalanced classes (scale_pos_weight). They recommend adjusting it as sum (negative instances)/sum (positive instances). Numbers of the surviving and non-surviving patients within the ICU for each age group are provided in the second and third columns of Table 1, respectively.

As the database was very large and the algorithms are computationally very expensive, the codes for fitting and running the model were been executed via GPU, an NVIDIA TESLA A100, under CUDA [19], decreasing the execution time compared to CPU-only architectures by a ratio of 2.2.

The set of XGBoost hyperparameters [20] used in the four classifiers is shown in Table 3. To identify the best performance in terms of higher AUROC, some of them (learning rate, subsample ratio, gamma, learning object, number of estimators, alpha region, and maximum depth of tree) were tuned using the grid search methodology. The set of XGBoost’s hyperparameters used in the four classifiers is shown in Table 3.

After completing the training phase, the test phase was carried out using 20% of the previously split data. The evaluation was done in terms of the most frequently used statistic parameters, namely AUROC, Precision, Specificity, Recall, and Accuracy. The results obtained are presented and discussed in Section 4.

### 3.4. Explainable Machine Learning Based on SHAP Analysis

Once the models were trained and predictions obtained, it became possible to proceed with the analysis phase using the already-selected SHAP. This permitted us to identify the features that had the highest impact on mortality prediction for each age group. In addition, it also allowed the threshold values at which a variable becomes harmful to the patient’s life to be determined. To do this, it was necessary to use each of the trained models and their corresponding test sets to obtain a series of graphs, which are explained in detail in Section 4. Once this analysis was carried out, the results could be used to identify the most important features to be displayed on the patient’s monitor and to define the threshold values at which the alarm system must warn healthcare personnel that the patient’s condition is dangerously worsening.

## 4. Results

The results of this work are presented in this section mainly in two aspects, namely, the quality of the classifier for mortality prediction among ICU patients, and SHAP analysis of the involved features. These two approaches allow the validity of the proposed method for identifying the features included in ICU monitoring systems to be evaluated.

### 4.1. Mortality Prediction Outcomes

As described Section 3, the variable to be predicted is the mortality of patients within the ICU, using XGBoost and a set of 129 features, for each age group. In order to quantify the quality of each XGBoost model, the corresponding AUROC, Accuracy, Precision, Specificity, and Recall metrics [21] have been obtained using the test subset by splitting the dataset into training and test subsets with an 80/20 ratio, then repeating the experiment five times with random splits in each repetition. The results of the average values of such evaluation metrics for each age group are provided in Table 4. In addition, results using all age groups as a unique dataset (*X_T_*) are also included.

It is noticeable that the values of these metrics are within the range of the current state of the art [22]. Nevertheless, the predictions in this work are only one step towards the final objective, namely the identification of the threshold values at which a health variable is considered critical to the patient, permitting the setting of useful alarms that can improve patient care.

**Figure 2:**
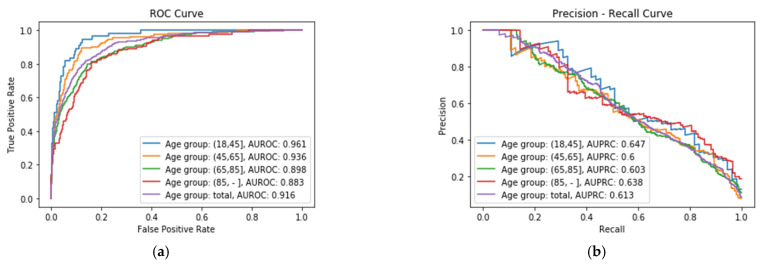
(**a**) ROC Curve obtained for each age group; (**b**) Precision–Recall Curve obtained for each age group.

Regarding the meaning of the metrics, specificity refers to the rate of survivors in the dataset being correctly identified as survivors, while the recall (also called sensitivity) refers to the rate of non-surviving patients in the dataset correctly identified as non-survivors. The specificity values are high for each age group (near 1) and the recall (sensitivity) is lower (near 0.6). This is due to the fact that the variable/class in the dataset used to make the predictions, namely the mortality, is very unbalanced (only 2930 non-survivors in a total of 36,693 surviving patients). It is also remarkable that the metrics obtained by splitting the dataset by age groups outperform, in general, the results obtained considering all the age groups as a unique dataset. Figure 2a shows the ROC Curve of each XGBoost classifier *C_i_* for the four age groups, where it is possible to see that the best performance is obtained for X_A_ group, with an AUROC value of 0.961, whereas the worst performance is obtained for X_D_ age group, with a value of 0.883. Figure 2b shows the Precision–Recall Curve of each age group, where it can be seen that the best results are obtained for the same age group, X_A_, with an Area Under the Precision–Recall Curve (AUPRC) value of 0.647.

### 4.2. SHAP Outcomes

After fitting each model *C_i_*, it is possible to proceed with explanation using SHAP, which permits identification of the features with the highest impact (features importance) on the prediction of mortality, as well as of the threshold values for alarms.

#### 4.2.1. Features Importance

**Figure 3:**
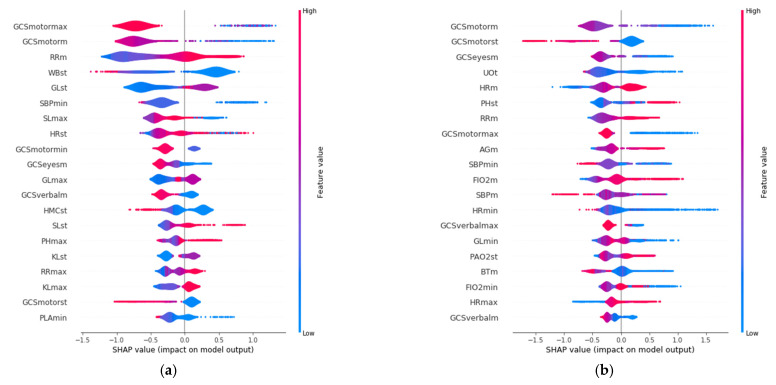

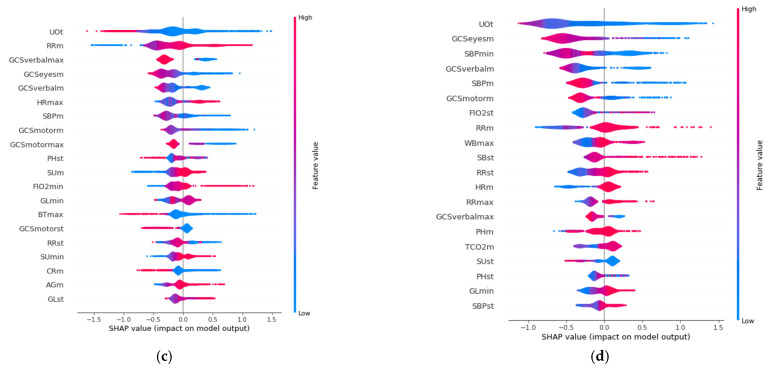
Features with the highest impact on the prediction of mortality for each age group: (**a**) 18–45 years; (**b**) 45–65 years; (**c**) 65–85 years; (**d**) over 85 years.

SHAP allows identification of the most convenient features to be monitored for each age range group based on the SHAP value corresponding to each feature value. The results of this analysis for the 20 variables with the highest impact on mortality for each age group are displayed in Figure 3, in decreasing order. In addition, the color scale denotes whether the value corresponds to a high or low value of the feature. For example, in the case of GCSmotormax (Maximum value of Motor Glasgow Coma Scale), it can be observed (Figure 3) that there is an impact on survival when the value is high (red color). That is, a patient with a high value in this feature would be more likely to survive than another with a lower value. It was observed that the list of features with the highest impact when predicting mortality are different for each age group.

The three features with the highest impact for the age group between 18 and 45 years (Figure 3a) are the maximum value of Glasgow Coma Motor Scale (GCSmotormax), the mean value of the Glasgow Coma Motor Scale (GCSmotorm), and the mean respiratory rate (RRm). For the 45–65 age group (Figure 3b), they are the mean value of the Glasgow Coma Motor Scale (GCSmotorm), the standard deviation of the Glasgow Coma Motor Scale (GCSmotorst), and the mean value of the Glasgow Coma Eyes Scale (GCSeyesm). For the 65–85 age group (Figure 3c), they are the total urine volume (UOt), mean breathing rate (Rrm), and the maximum value of Glasgow Coma Verbal Scale (GCSverbalmax). Finally, the three most important features for the group over 85 years old (Figure 3d) are the total volume of urine (UOt), the mean value of the Glasgow Coma Eyes Scale (GCSeyesm), and the minimum value of systolic blood pressure (SBPmin).

#### 4.2.2. Threshold Values Identification

**Figure 4:**
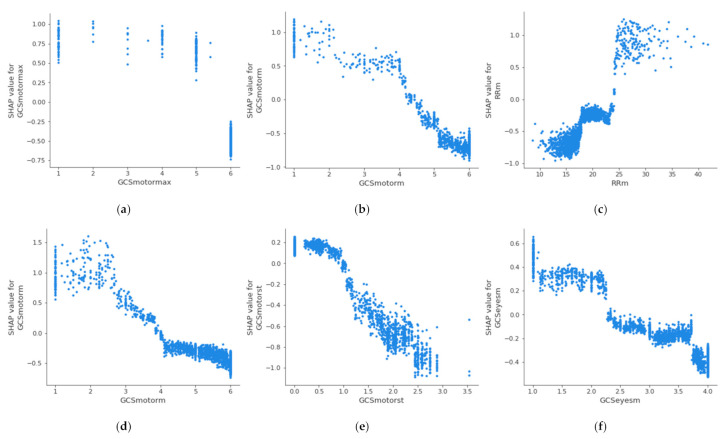

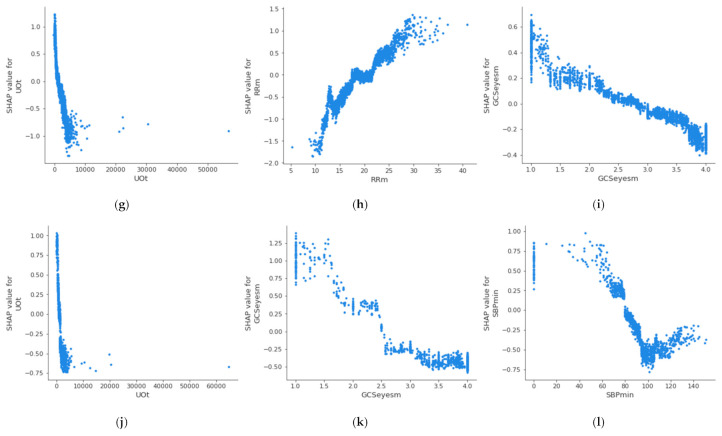
Dependence plot of the three main features for each age group: (**a**–**c**): 18–45 years; (**d**–**f**): 45–65 years; (**g**–**i**): 65–85 years; (**j**–**l**): over 85 years.

For the creation of alarms, it is of interest to know the threshold value at which a health variable is critical for the patient’s health. Figure 4 shows the partial dependence plot of the three health features that have the highest impact on mortality in each age group, which allows the determination of the above-described threshold values at which the feature becomes dangerous for the patient. A feature has an impact on mortality when its SHAP value (abscissas in Figure 4) is greater than 0. On the other hand, if the SHAP value is less than 0, it has an impact on the patient’s survival. Therefore, the threshold value at which set an alarm for a specific feature is the value at which its SHAP value is 0. The methodology for the identification of these threshold values using SHAP is automatic and generalized and can be applied to different sets of features, age groups, or classifiers. The partial dependence plots show the marginal effect that one or two features have on a predicted outcome in the machine learning model; this allows to configuration of the alarm system to warn health personnel.

In the case of the age group between 18 and 45 years, it can be observed that when the maximum value of the Glasgow Coma Motor Scale (A) is less than 6, it begins to be critical for the patient’s health. In the case of the mean value of the Glasgow Coma Motor Scale (B), this happens when it is less than 4.2. In the case of the mean respiratory rate (C), it begins to be critical for the patient’s health when it is greater than 24 bpm. Table 5 shows the threshold values for the three features with the highest impact on mortality for each age group; it can be observed that these differ between the groups. The threshold of the mean value of respiratory rate varies from 24 bpm in X_A_ age group to 18 bpm in X_C_ age group; in the case of the mean value of the Glasgow Coma motor scale, it is different in the X_A_ age group (4.2) and X_B_ age group (3.8); finally, the threshold of the mean value of the Glasgow Coma eyes scale changes between the X_B_ age group (2.5), X_C_ age group (2.8), and X_D_ age group (2.6). This suggests that performing this analysis by age group leads to a more precise alarm configuration. The same analysis could be performed for the remaining features, both in this age group and in the rest of the age groups.

## 5. Discussion

The results show that the performance of the classifier that predicts mortality in ICU patients using XGBoost is equal to or better than other machine learning techniques in the current state of the art. The highest AUROC value, 0.961, was obtained for the age group X_A_:(18, 45]. However, it must be taken into account that it is complex to make this comparison. There are a large number of studies that analyze the mortality of patients within the ICU; however, none of those consulted performed the prediction by age groups in a generic way. In this work, the dataset was split into four groups, reducing the amount of data to train each of the four classifiers, resulting in lower values for the evaluation metrics than what would be obtained with a non-split dataset. This can be seen in Table 4, where X_T_ row refers to the results obtained without splitting by age groups.

The explainability of the model using SHAP includes the identification of the features with the highest impact for each age group, as well as of the thresholds for each feature and each age group (that is, the value at which the a health variable begins to be critical for the patient’s health). As mentioned above, no studies were found that analyzed the problem of mortality within the ICU by age group. There are studies focused on specific diseases [15,23], but the features that most affect mortality and the thresholds involving a specific disease may be different from those that affect mortality in a general way. In addition, there are other factors that affect the results, such as the database, the selected variables, the predictor model, the data collection time window, or the defined age groups, among others. It can be observed how the features threshold at which the value of a health variable is considered critical to the patient vary depending on age group.

As previously indicated, the idea was to improve monitoring systems for the adult ICU; hence, patients under the age of 18 were not part of the study. Therefore, the analysis carried out is only valid for the adult ICU; however, the same methodology could be followed for application in the pediatric ICU.

## 6. Conclusions

This article introduces a new methodology to improve ICU monitoring systems with an age-based stratification approach, automatically identifying threshold values of the most important clinical variables to be monitored by using XGBoost classifiers and SHAP techniques for explainable machine learning. Results that confirm the usability of the proposed methodology are provided using the MIMIC-III database. The methodology consists of three main steps: dataset pre-processing in the form of splitting by age groups, model fitting, and analysis using SHAP. The method has been evaluated using standard evaluation statistics and analyzed using the well-established SHAP technology.

The results show that the model is able to predict patient mortality in the ICU with AUROC values of 0.961, 0.936, 0.898, and 0.883 for the age groups X_A_ (18–45), X_B_ (45–65), X_C_ (65–85), and X_D_ (85+), respectively. Moreover, by using SHAP it can be observed how the features threshold at which the value of a health variable is considered critical to the patient vary depending on age group, which justifies the division by age group instead of the computation of generic thresholds. In addition, the results obtained by the predictor are generally better when following the age-based approach than the generic.

This methodology can be extended in several ways in the future. Possible modifications include using another type of predictor model instead of XGBoost, another prediction variable instead of mortality (e.g., sepsis), selecting a different set of clinical variables from the 33 proposed in this study, using a time slot different from 24 h for obtaining features, or defining different age groups. Those new approaches would provide different results, but the same proposed methodology could be used.

## Figures and Tables

**Table 1:** Age groups and survival information.

Age Group	Total Number of Patients	Survivors	Non-Survivors
X_A_: (18, 45]	5447	5194	253
X_B_: (45, 65]	12,370	11,604	766
X_C_: (65, 85]	15,019	13,609	1410
X_D_: (85, ∞)	3857	3356	501

**Table 2:** Features extracted from clinical variables.

Clinical Variable	Total	Maximum	Minimum	Average	Standard Deviation
Urine output	X				
Glasgow Coma Motor Scale		X	X	X	X
Glasgow Coma Eyes Scale		X	X	X	X
Glasgow Coma Verbal Scale		X	X	X	X
Systolic blood pressure		X	X	X	X
Heart rate		X	X	X	X
Body temperature		X	X	X	X
PaO_2_		X	X	X	X
FiO_2_		X	X	X	X
Serum urea nitrogen level		X	X	X	X
White blood cells count		X	X	X	X
Serum bicarbonate level		X	X	X	X
Sodium level		X	X	X	X
Potassium level		X	X	X	X
Bilirubin level		X	X	X	X
Respiratory rate		X	X	X	X
Glucose		X	X	X	X
Albumin		X	X	X	X
Anion gap		X	X	X	X
Chloride		X	X	X	X
Creatinine		X	X	X	X
Lactate		X	X	X	X
Calcium		X	X	X	X
Haematocrit		X	X	X	X
Haemoglobin		X	X	X	X
INR		X	X	X	X
Platelets		X	X	X	X
Prothrombin Time Test		X	X	X	X
Activated Partial Thromboplastin Time		X	X	X	X
Base excess		X	X	X	X
PaCO_2_		X	X	X	X
Total CO_2_		X	X	X	X

**Table 3:** Hyperparameters used in the four classifiers.

Hyperparameter Name	Hyperparameter Value
Learning rate	0.01
Subsample ratio of columns	0.4
Gamma parameter	1
Learning object	Logistic regression
Number of estimators	1000
Alpha region	0.3
Maximum depth	3
Minimum loss reduction	0
Minimum sum of instances	1
Maximum delta step	0
Method to sample	Uniform
Regularization term	1
Updated tree parameter	True
Normal boosting process	True
Maximum number of nodes	0
Maximum number of discrete bins	256
Number of parallel trees	1

**Table 4:** Mortality prediction outcomes (Metrics).

Age Group	AUROC	Precision	Specificity	Recall	Accuracy
X_A_: (18, 45]	0.961	0.566	0.998	0.545	0.956
X_B_: (45, 65]	0.936	0.518	0.966	0.570	0.941
X_C_: (65, 85]	0.898	0.533	0.946	0.571	0.909
X_D_: (85, ∞)	0.883	0.598	0.943	0.462	0.869
X_T_: (18, ∞)	0.916	0.444	0.925	0.683	0.905

**Table 5:** Features and their thresholds values for mortality prediction in the four age groups.

Age Group	Rank	Feature	Threshold
X_A_: (18, 45]	1	GCSmotormax	6
	2	GCSmotorm	4.2
	3	RRm	24 bpm
X_B_: (45, 65]	1	GCSmotorm	3.8
	2	GCSmotorst	1
	3	GCSeyesm	2.5
X_C_: (65, 85]	1	UOt	1000 mL
	2	RRm	18 bpm
	3	GCSeyesm	2.8
X_D_: (85, ∞)	1	UOt	1000 mL
	2	GCSeyesm	2.6
	3	SBPmin	80 mmHg

## Data Availability

By reasonable request to José A. González-Nóvoa.
